# The Role of Small-Bowel Endoscopy in the Diagnosis and Management of Small-Bowel Neuroendocrine Tumours

**DOI:** 10.3390/jcm13226877

**Published:** 2024-11-15

**Authors:** Elisabet Maristany Bosch, Faidon-Marios Laskaratos, Mikael Sodergren, Omar Faiz, Adam Humphries

**Affiliations:** 1 St Mark’s National Bowel Hospital, Acton Lane, Park Royal, London NW10 7NS, UK; elisabet.bosch@nhs.net (E.M.B.); adam.humphries@nhs.net (A.H.); 2 Department of Surgery and Cancer, Faculty of Medicine, Hammersmith Hospital, London W12 0TS, UK; mikael.sodergren@nhs.net; 3 Imperial Neuroendocrine Tumour Unit, ENETS Centre of Excellence, London W12 0TS, UK; 4 Imperial College London, London SW7 2AZ, UK; omar.faiz@nhs.net; 5 Department of Surgery, St Mark’s National Bowel Hospital, Acton Lane, Park Royal, London NW10 7NS, UK

**Keywords:** small-bowel neuroendocrine tumours, video capsule endoscopy, double-balloon enteroscopy

## Abstract

Neuroendocrine tumours (NETs) are relatively rare neoplasms but represent one of the most frequent types of primary small-bowel tumours. Their incidence is rising, and this is most likely because of their more frequent early-stage detection, physician awareness, and increasing availability and use of imaging and small-bowel endoscopic techniques, such as video capsule endoscopy and device-assisted enteroscopy, which enable the detection, localisation, and histological sampling of previously inaccessible and underdiagnosed small-bowel lesions. This review summarises the role of small-bowel endoscopy in the diagnosis and management of small-bowel NETs to assist clinicians in their practice. Small-bowel endoscopy may play a complementary role in the diagnosis of these tumours alongside other diagnostic tests, such as biomarkers, conventional radiology, and functional imaging. In addition, small-bowel enteroscopy may play a role in the preoperative setting for the identification and marking of these tumours for surgical resection and the management of rare complications, such as small-bowel variceal bleeding, in cases of portal hypertension due to the encasement of mesenteric vessels in fibrotic small-bowel NETs.

## 1. Introduction

Neoplasms of the small intestine are relatively rare, especially considering the length of this organ. The two most common types of small-bowel neoplasms are neuroendocrine tumours (NETs) and small-bowel adenocarcinomas (SBAs), with other neoplasms, such as gastrointestinal stromal tumours, sarcomas, and lymphomas, seen less frequently [1,2].

Currently, NETs are the most frequent type of primary small-bowel tumours, with an incidence that has been rising over the past four decades [2,3]. In the United States, from 1985 to 2005, the proportion of newly diagnosed primary small-bowel tumours diagnosed as NETs increased from 28% to 44%, overtaking SBAs as the main primary tumour type, which proportion dropped from 42% to 33% in the same time period [2]. A large population-based study performed in the United States with data from the Surveillance, Epidemiology, and End Results (SEER) program, showed that the annual incidence of NETs increased from 1.09 per 100,000 in 1973 to 6.98 per 100,000 in 2012 (a 6.4-fold increase). The study also showed an increase in overall survival over time, especially pronounced in distant gastrointestinal NETs and in distant pancreatic NETs, reflecting improvements in systemic therapies and the multidisciplinary management and centralised care of these advanced NETs [3]. Other studies, including large European and Canadian studies, support these findings, with an overall increase in the incidence of NETs reported [4,5]. This steady increase in incidence is most likely because of a combination of factors, including increased physician awareness and more widespread use of small-bowel imaging and small-bowel endoscopic techniques, namely, video capsule endoscopy (VCE) and device-assisted enteroscopy (DAE), which enable earlier detection, localisation, and histological sampling of previously inaccessible and undetectable small-bowel lesions. With improvements in management, overall survival has also improved, resulting in a higher prevalence of patients with small-bowel NETs in recent years.

### 1.1. Pathology and Grading

Neoplasms with proven neuroendocrine differentiation (NENs) are defined as expressing biomarkers of the two regulated pathways of the secretion of normal neuroendocrine cells or neurons, specifically those associated with large, dense core vesicles (LDCVs) and small synaptic-like vesicles (SSVs); more recently, they have also been recognised to express zinc finger transcription factor insulinoma-associated 1 (IA1 or INSM1) [6,7]. Though they all share some common features, they can have very diverse biochemical properties related to the organ of origin, with significantly different natural histories, as well as malignant potentials. The last revised *2022 WHO Classification for Neuroendocrine Neoplasms* proposed a universal definition system for neuroendocrine neoplasia based on the degree of morphological differentiation and proliferative grading to standardise the nomenclature and facilitate the classification of these lesions [7]. NETs are defined as well-differentiated epithelial neoplasms composed of tumour cells that faithfully retain the morphological and molecular features of normal neuroendocrine cells, with a slow to moderate growth pattern. NETs are generally graded as G1, G2, and G3 based on their mitotic rate and Ki67 proliferation index, and this is associated with their prognosis and potential for metastasis [1,7]. In contrast, poorly differentiated neuroendocrine carcinomas (NECs) tend to have a more aggressive behaviour, grow rapidly, and are, by definition, high grade [1,7].

### 1.2. Location and Clinical Presentation

Gastroenteropancreatic NETs are most commonly found in the small intestine (45%), rectum (20%), appendix (16%), colon (11%), pancreas (5–10%), and stomach (7%). The ileum is the most common site for small-bowel NETs, contrary to adenocarcinomas, which are most commonly found in the duodenum [2,3,8]. The specific term “small-bowel NETs” includes those lesions that arise from the ligament of Treitz to the ileocecal valve. This definition does not include duodenal neoplasms, which are biologically and clinically distinct from tumours that arise in the jejunum or ileum [1,9,10]. Therefore, duodenal NETs will not be reviewed in this article.

At the time of the diagnosis, around 30% of patients have metastatic disease, and up to 40% have regional lymph node involvement [8]. Symptoms vary depending on the tumour functionality and location of the lesions. NETs of the jejunum and ileum usually initially present as non-functioning tumours but, in the presence of liver metastases at later stages, may manifest with carcinoid syndrome [11,12,13]. Abdominal pain is a frequent, early, non-specific symptom, and other early symptoms can be related to gastrointestinal bleeding and anaemia. Primary tumours, despite their small size, may cause an extensive fibrotic reaction in the small bowel involving the lymph nodes and mesentery, leading to potential mesenteric ischemia and small-bowel obstruction [13,14,15]. This desmoplastic reaction can also involve retroperitoneal organs, which can potentially cause obstructive uropathy and hydronephrosis. Carcinoid syndrome is rarely seen in the absence of metastatic disease and is caused by excessive secretion of endogenous substances, most frequently serotonin, neurokinin A, histamine, and others [8,9,12]. Patients can present with facial flushing, diarrhoea, abdominal cramps, heart valve disease (carcinoid heart disease), telangiectasia, wheezing, and oedema, in the order of decreasing frequency [12,14,16]. Complications related to non-cirrhotic portal hypertension, such as ascites and varices, are more likely related to advanced neoplastic liver disease, when the liver parenchyma is replaced with NET metastases, and heart failure due to carcinoid heart disease, rather than a direct excess of hormone secretion. In addition, encasement of the mesenteric vessels by the mesenteric fibrotic mass can also lead to the development of ascites and small-bowel varices [9,12].

The slow growth and small size of these tumours do not always correlate with a better prognosis. As previously stated, 30% of patients have metastatic disease at the time of the diagnosis, and 40% have regional lymph node involvement. Usually, at this stage, small-bowel NETs are commonly >2 cm in size, with invasion of the muscularis propria, and up to 40% can present as multifocal tumours. In contemporary surgical series, the incidence of multifocal small-bowel NETs is as high as 50%. Familial forms of small-bowel NETs appear to have an even higher rate of multifocal disease, in the range of 80%. Contrary to what was previously thought, recent data suggest that multifocal disease might not be associated with a poorer prognosis [17,18,19].

### 1.3. Treatment Strategies

Management of small-bowel NETs requires a multidisciplinary team (MDT) approach. Assessment of symptoms with special focus on carcinoid syndrome is important. All localised resectable small-bowel NETs should be operated on if possible because this is the only chance for long-term cure. In the presence of advanced unresectable small-bowel NETs with G1 or G2, long-acting somatostatin analogues (SSAs) are the preferred first-line treatment. Watch and wait may be an option in selected patients (G1, asymptomatic, low tumour burden, and/or stable disease). In patients with advanced disease and somatostatin receptor negative disease, interferon alfa (IFNα) or everolimus can be used as the first-line treatment if locoregional therapy is not an appropriate option. Chemotherapy plays a minor role in highly selected patients (especially NET G3 and/or rapid tumour progression).

The recommended second-line treatment in the presence of somatostatin receptor positive tumours is PRRT, followed by everolimus. Rechallenge PPRT and high-dose SSAs may be considered in selected scenarios. Chemotherapy is rarely recommended for small-bowel NETs unless they are NET G2 with higher Ki-67 indices (15–20%) or NET G3 with rapid tumour progression.

In patients with small-bowel NET G1/G2 stage IV disease and exclusive liver metastases, a surgical approach is indicated if complete (R0) resection can be achieved. In non-surgical candidates, locoregional liver-directed therapies should be explored in predominant liver disease.

In patients with refractory carcinoid syndrome and predominant diarrhoea, telotristat ethyl, which is an inhibitor of serotonin synthesis, should be added to SSAs. SSA dose escalation can be considered in patients with refractory carcinoid syndrome or, alternatively, the addition of IFN-α. Locoregional and ablative treatments, including debulking surgery, should be considered early for refractory carcinoid syndrome. PRRT is also an option, especially if progressive metastatic disease is present. Everolimus may be considered in individual patients in combination with SSAs if significant tumour growth is present [20].

## 2. Methodology

An extensive bibliographical search was performed in PubMed in order to identify relevant literature on the topic, including systematic reviews and prospective and retrospective studies, as well as the last updated clinical practice guidelines, using the following keywords: “small-bowel neuroendocrine tumours”, “video capsule endoscopy” and “double-balloon enteroscopy”. The reference lists from the selected studies were manually examined to identify further relevant reports, and a total of 56 studies were finally included in this narrative review.

## 3. Diagnosis

It remains a challenge to detect, localise, and diagnose tumours in the small bowel; however, these are essential tasks in order to offer an appropriate treatment and management plan. The lack of specific early symptoms and challenge in accessing the small bowel often delay the final diagnosis. Common radiological techniques, such as CT and MRI, either via a standard technique or enterography in combination with enteroclysis, are usually used as a first-stage investigation in the presence of non-specific abdominal symptoms, although with a high risk for missing small-bowel lesions, especially those of smaller sizes [21,22]. PET-CT with 68Ga-DOTA peptides (somatostatin-receptor-based imaging) remains the most sensitive radiological modality for the detection of well-differentiated and low-grade NETs with somatostatin receptor expression, but unlike endoscopic techniques, it does not allow for the accurate localisation of lesions for surgical treatment or the obtention of histological samples, which is considered as the gold standard for a definite diagnosis; moreover, small lesions (<5 mm) might also be missed [23,24]. In the presence of metastatic disease, finding the primary tumour is considered as useful for definitive treatment, even for non-resectable disease, as medical therapy may vary depending on the primary site. However, in up to 5–10% of the cases after the discovery of metastatic and lymph node diseases, the primary tumour site still remains unknown, despite the use of multiple radiological techniques [22,25].

The introduction of non-invasive and invasive endoscopic techniques, such as VCE and DAE, and especially double-balloon enteroscopy (DBE), has facilitated the localisation and diagnosis of single and multifocal lesions via histological sampling of previously inaccessible or incidental small-bowel NETs. Accurate data regarding the efficacy of VCE and DBE in the detection of SB NETs are lacking, although published data from other small-bowel lesions have reported good diagnostic outcomes for both VCE and DBE, with DBE having the additional advantage of histological sampling and marking with tattoos [26].

## 4. Video Capsule Endoscopy

**Figure 1:**
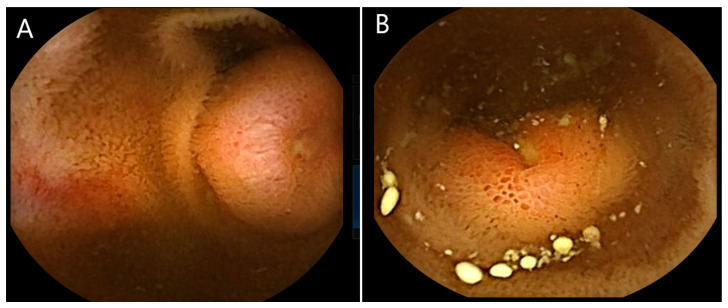
VCE appearances of small-bowel NETs (**A**,**B**).

Since its introduction in 2001, VCE has marked a new era for investigating the small intestine as a safe, effective, and non-invasive technique. It is now well established as a primary diagnostic modality for the investigation of obscure gastrointestinal bleeding, iron deficiency anaemia (IDA), suspected Crohn’s disease in the absence of bowel obstruction or stenosis, abnormal imaging findings and clinical symptoms in the presence of normal imaging results, as well as the assessment of established Crohn’s disease, surveillance of polyposis syndromes, and diagnosis of small-bowel tumours (SBTs) [27]. Small-bowel NETs usually appear in VCE videos as submucosal lesions, sometimes with superficial ulceration (Figure 1). They may be solitary lesions, but, occasionally, multifocal tumours may be seen.

As stated in the latest ESGE guidelines, small-bowel VCE is recognised as a useful tool for the surveillance of polyposis syndromes, as well as suspected SBTs, in patients at increased risk of an SBT [28,29]. The American Society for Gastrointestinal Endoscopy also states that VCE is indicated for the surveillance of polyposis syndromes and the investigation of suspected SBTs [30]. If a tumour is strongly suspected, other examinations should also be considered [28] because small-bowel VCE may produce false-negative results in segments of rapid intestinal passage, such as the proximal small intestine [31,32,33]. In the US, it is recommended to perform CT or MRI for the initial investigation of small-bowel tumours, followed by VCE if not detected or when further information is needed [30,34]. The most recent (2017) Japanese practice guidelines advised the combined use of VCE and contrast-enhanced CT for screening small-bowel tumours, with VCE especially recommended for its high sensitivity in the detection of flat and small (≤10 mm) lesions, including SB NETs [31].

Because of the rarity of SB tumours in general, there are no prospective studies available, and most of the current data are based on retrospective studies for other small-bowel conditions, such as obscure gastrointestinal bleeding. A retrospective UK study, performed by Frilling et al. to assess the role of VCE in locating primary SB NETs when conventional imaging failed to identify the primary lesion, reached the conclusion that VCE is a useful diagnostic tool and should be considered as a part of the diagnostic workup in selected patients presenting with metastatic neuroendocrine tumours of unknown primary locations. The study included 390 patients with metastatic NETs, and in 11 (2.8%), the location of the primary tumour was unknown. The study showed that in patients where the primary tumour could not be found using conventional radiology, VCE identified small-bowel NETs that were later confirmed histologically in eight out of ten patients who managed to swallow the capsule and underwent surgery [35]. Another study by Furnari et al. assessed the diagnostic yield of VCE compared to surgical exploration in patients that had metastatic NETs with unknown primary tumours and inconclusive first-line investigations; VCE had 75% sensitivity and 38% specificity compared to exploratory surgery for the detection of the primary tumour. Eleven patients out of sixteen who agreed to undergo surgery had a positive VCE for suspected small-bowel NETs, and out of the sixteen patients, laparotomy was able to find the primary tumour in 11/16 patients: seven subjects had small-bowel NETs; three, pancreatic NETs; and one, a well-differentiated biliary NET. One of the seven patients with primary small-bowel NETs had three multifocal jejunal tumours; therefore, a total of nine small-bowel NETs were diagnosed by surgery. The authors recommended that VCE may provide additional information when conventional investigations are inconclusive about the primary site in the setting of metastatic gastroenteropancreatic NETs [36]. A comparison of small-bowel VCE with CT, enteroclysis, and nuclear imaging (with Octreoscan and MIBG scintigraphy), for the detection of small-bowel lesions, showed that CT and enteroclysis did not detect primary small-bowel NETs; nuclear imaging demonstrated abnormalities in the abdominal area in thirteen patients but was unable to relate this to an intestinal localisation, and VCE revealed small-bowel tumours in nine patients, showing a high diagnostic yield (45%) in identifying primary tumours. These were confirmed histologically as NETs in five out of seven patients who underwent surgery [37]. Contemporary data show that the incidence of multifocal tumours of small-bowel NETs is as high as 50%, and familial forms of small-bowel NETs appear to have an even higher rate of multifocal disease, at up to 80%. The burden of the disease can be underestimated by VCE, with DBE allowing for a better estimation of the disease burden [19,38]. Identification of multifocal disease is important in order to offer adequate management and prognostication, even though recent data suggest that multifocal disease might not be associated with a poorer prognosis [17,18,19].

Although data suggest that VCE is a good modality for detecting small-bowel NETs, a miss rate of SBTs has been documented. Submucosal lesions, such as GI stromal tumours and some NETs where the tumour is not disrupting the overlying mucosa (and, therefore, has a complete submucosal appearance), can be easily missed because of their minimal endoluminal component. An overall higher miss rate has been reported in the proximal small bowel compared to the distal small bowel. The VCE diagnostic yield of small-bowel tumours in the distal duodenum is significantly lower than that at other sites, probably because of the rapid intestinal passage more often seen in the proximal bowel [39]. Another study also showed that missed tumours were mostly located in the proximal small bowel, with an overall miss rate of 16.5% [40]. The quality of the bowel preparation decreases as the capsule reaches the distal small bowel and, so, could contribute to a lower detection rate of distal lesions.

The main adverse event associated with VCE is capsule retention, potentially requiring subsequent endoscopic or surgical retrieval. In the setting of SBTs, the rate of capsule retention has been described as from 6 to as high as 21% [41,42]. However, there are no data exclusively for NETs, and retention is likely much lower in NETs, as small-bowel obstruction is an unusual initial presentation, although retention can occur in the setting of extrinsic compression due to metastatic disease or adhesions. In the case of bowel obstruction due to capsule retention, surgery is usually recommended, which allows for both capsule retrieval and tumour resection [42]. If a small-bowel stricture or obstruction is suspected, before considering VCE, the use of a patency capsule is recommended [43].

Once the lesions are identified via VCE or are highly suspected, a further invasive procedure, such as DBE, is the next step in order to confirm the findings, obtain histological confirmation, and mark the small-bowel location with a tattoo to aid potential surgical resection.

## 5. Double-Balloon Enteroscopy

**Figure 2:**
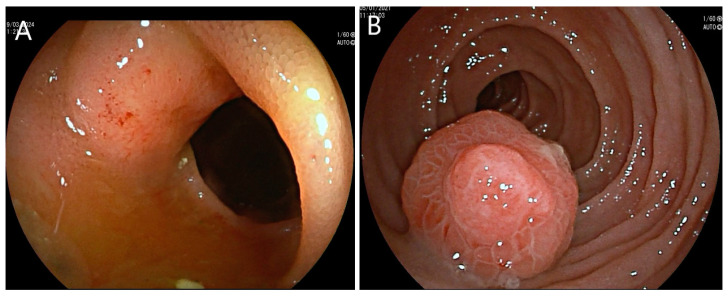
DBE images of small-bowel NETs (**A**,**B**).

DAE is an endoscopic technique that enables the determination of the precise location and number of tumours and allows for the histological sampling of small-bowel lesions that were previously inaccessible and/or in need of invasive surgical procedures. Most of the literature on small-bowel enteroscopy relates to the DBE technique. Figure 2 shows some representative images of small-bowel NETs diagnosed by DBE.

The route of choice, oral (antegrade) or anal (retrograde), is guided by findings of previous diagnostic investigations. If the location of the small-bowel lesion is unknown or uncertain, ESGE guidelines recommend that the antegrade route should be taken initially, with the possibility to combine this with the retrograde route if no lesion is found, preferably in a different session. With a combined oral and anal approach, a complete small-bowel examination can be achieved in up to 86% of patients [44]. Similar to VCE, studies of DBE in relation to SB NETs are scarce.

In a retrospective study that reviewed 85 patients with primary small-bowel NETs, the authors assessed different imaging techniques (CT, MRI, or somatostatin receptor imaging (SRI)) and double-balloon enteroscopy to identify the primary tumour. The study showed that DBE was significantly more accurate at identifying the primary NET compared to CT, MRI, or SRI. The sensitivity of each in identifying small-bowel NETs was 59.7% for CT, 54% for MRI, 56% for SRI, and 88.1% for DBE. A total of 18 (21.2%) patients had primary tumours not identified on imaging, of which 13 underwent DBE, and 12 of the 13 (92.3%) DBEs identified the primary lesion. DBE was also shown to be significantly better in detecting multifocal lesions when compared to CT and somatostatin receptor imaging but not MRI [45]. Another retrospective study comparing multifocal versus solitary NETs, to examine the associated outcomes, had, as a secondary aim, the evaluation of the utility of DBE and capsule endoscopy. Of the 85 patients included in the study, 46 (54.1%) had multifocal disease, of whom 37 (80.5%) had an ileal primary tumour. DBE was able to detect additional lesions in 28 patients (62.2%) compared to VCE, of which 23 (82.1%) had NET confirmed based on pathology. No differences in survival or recurrence after surgical resection were seen for multifocal versus solitary disease [19]. A single-centre Italian study aimed to report the efficacy of DBE for the detection of small-bowel NETs. A total of 25 patients were referred with suspected small-bowel NETs; after an extensive workup, six patients underwent DBE, which was diagnostic in three patients with ileal NETs. The group reported an overall sensitivity and specificity of 60% and 100%, respectively, showing that DBE is a safe and effective procedure in diagnosing SB NETs and for tattoo placement prior to surgery. It was also suggested that DBE should be preferred over VCE in the presurgical setting, given its high specificity [46].

A similar prospective study by Bellutti et al. aimed at evaluating modalities for the detection of primary small-bowel NETs, included 12 patients with suspected NETs with carcinoid syndrome (either with liver metastasis or symptomatic with elevated tumour markers) or obscure gastrointestinal bleeding, who underwent CT scan, OGD, ileocolonoscopy, and octreotide scintigraphy prior to DBE. Capsule endoscopy was also performed on four patients. These investigations failed to identify the primary tumour. A total of 17 DBEs were then performed on these 12 patients; a submucosal lesion of the ileum or jejunum was detected in seven patients (53%), and in four, this finding was confirmed by the surgical resection of an NET, showing a diagnostic yield for DBE of 33%. In two patients, surgery did not confirm the tumour, thus resulting in a 17% false-positive rate [47]. In the study, DBE biopsies were unable to provide evidence of NETs in any of the patients with a visible tumour; possible explanations for this may be the use of small-sized forceps during DBE and the submucosal origin of the tumour. In light of these results, the study concluded that DBE was not as advantageous for detecting small-bowel NETs as previously thought, and, therefore, with the current available evidence, DBE should only be performed in selected cases, possibly based on a positive previous workup. It is worth noting, however, that the study was limited by few patients, which highlights the need for larger and, ideally, multicentre studies to evaluate clinical outcomes of DBE in the management of small-bowel NETs [47].

The ESGE guidelines recommend the consideration of DBE in preference to VCE if imaging tests have already demonstrated a suspected small-bowel tumour, as its diagnostic yield is superior. In this setting, DBE may allow for biopsies for histological analysis and localisation of the lesion for future surgical resection via tattoo placement. Moreover, when a subepithelial mass is detected by VCE, DBE is recommended to confirm the diagnosis, as NETs can sometimes show this appearance. The Japanese guidelines state that DBE is an excellent technique to detect and diagnose small-bowel lesions, as the diagnostic yield of biopsy samples has been shown to be high for epithelial tumours, malignant lymphoma, and neuroendocrine tumours (NETs), although lower for metastatic small-bowel tumours and GIST [31].

Small-bowel NETs have a high incidence of multifocality; because of this, DBE can be used in preoperative assessments to detect multifocal NETs and place a tattoo in order to plan the surgical approach and extent of the bowel resection [19]. In a retrospective study of patients who had undergone small-bowel resection, DBE prior to surgery identified additional lesions in 54% of the patients compared to 18% with VCE [19]. Although DBE seems to be a good technique to identify multifocal lesions, some centres have limited access to DBE. The North American Neuroendocrine Tumour Society (NANETS) guidelines suggest that if access to DBE is limited, multifocal tumours may be most reliably found during surgery, by surgical exploration and examination of the small bowel by careful digital palpation [10].

The role of endoscopy in small-bowel NETs is still reserved for diagnostic proposes and not considered for therapeutic resection. Although DBE-assisted endoscopic mucosal resection (EMR) has proven to be a safe and effective technique for the removal of other small-bowel polypoid mucosal lesions [27], surgical resection remains the mainstay for jejunal and ileal NETs, as these lesions are submucosal and may be associated with invasion and lymphatic spread, usually requiring a more extensive surgical resection.

DBE is considered as a safe technique with very low risks of complications, even in the elderly. The most common complications reported are bleeding, perforation, and pancreatitis [27]. Bleeding and perforation are usually associated with therapeutic procedures, such as polypectomy and dilation of strictures, so they are rarely encountered in the diagnostic workup of small-bowel NETs [48,49]. Pancreatitis has been reported to occur in 0.3% of DBE procedures, most commonly in antegrade DBE, and is suspected to be related to ischemic/traumatic injury to the pancreas during push-and-pull manoeuvres. The duration and depth of the procedure, endoscopic experience, and balloon insufflation before the ligament of Treitz have been associated with the development of acute pancreatitis [48,49,50]. As stated in the ESGE guidelines, the risk of pancreatitis may be reduced by applying a careful atraumatic technique, minimising mechanical stress, and avoiding inflation of balloons within the proximal duodenum.

DBE should also be considered in less common situations related to small-bowel NET complications, such as bleeding [51]. Gastrointestinal bleeding can be an early symptom from the primary lesion. In addition, atypical small-bowel varices (SBVs), secondary to portal hypertension and mesenteric fibrosis/involvement of mesenteric vessels, are rare but have been described, usually in metastatic disease [9,12,52,53]. In this unusual situation, radiologic embolisation tends to be the first line of treatment; however, when this fails or is not available, DBE-facilitated cyanoacrylate injection endotherapy of SBVs appears to be a safe and effective option to achieve haemostasis. As shown in a retrospective review of DBE-facilitated cyanoacrylate injection endotherapy of SBVs, ten DBEs were performed on six patients with small-bowel varices from different aetiologies, one of which was secondary to a small-bowel NET, and where no radiologic or surgical options were deemed as feasible, thirteen nests of SBVs were identified and injected with cyanoacrylate glue without haemorrhagic or embolic adverse events [52].

## 6. Economic Aspects

Economic aspects of endoscopic procedures should also be taken into account to achieve a cost-effective diagnostic approach. To date, there have been few published data comparing costs of endoscopic techniques regarding diagnostic and therapeutic procedures on the small bowel, and there are currently no data focusing on costs for the diagnosis of small-bowel lesions, including NETs. There are scarce data available comparing procedural costs of VCE and DBE in diagnosing and treating small-bowel bleeding, as analysed by Wienke et al., in 2010, who reached the conclusion that in their patient cohort, the strategy for performing a diagnostic VCE first, instead of an initial diagnostic and therapeutic DBE in the same procedure, seemed to minimise procedural costs [54]. Another study published by Gerson et al., in 2008, explored the optimal management strategy for obscure GI haemorrhage using a cost-effective analysis. The study showed that an initial DBE approach would be cost-effective for patients with obscure bleeding. However, a diagnostic VCE-first strategy, followed by a targeted DBE, may be associated with better long-term outcomes because of the potential for fewer complications and decreased utilisation of endoscopic resources [55]. Therefore, further cost-effectiveness analyses are required for comparing not only both endoscopic techniques but also common radiological tests used routinely in the diagnosis of suspected small-bowel NETs.

In addition, the ESGE guidelines recommend the consideration of DBE in preference to VCE if imaging tests have already demonstrated a suspected small-bowel tumour, as its diagnostic yield is superior. In this setting, DBE may allow for biopsies for histological analysis and localisation of the lesion for future surgical resection via tattoo placement. Although no data have been published on the economic value of this approach in the setting of small-bowel NETs, it is expected that there would be considerable cost savings by not additionally performing a VCE to visualise a small-bowel lesion that has been confirmed based on previous radiology imaging, if this is not required, and by proceeding directly to a DBE for tissue diagnosis in this setting.

## 7. Conclusions

In summary, since the introduction of small-bowel endoscopic techniques, the detection and diagnosis of small-bowel NETs have been facilitated with the use of VCE and DBE as minimally invasive, safe, and effective diagnostic tools. In addition, DBE plays a role in the preoperative setting, as it allows for tattoo marking at the tumour site, in both solitary tumours and multifocal disease, in order to guide surgical resection. Currently, endoscopic resection is not indicated for the management of localised small-bowel NETs because of their submucosal origin within the thin small-bowel wall and the risk of deep and extraintestinal invasion, which requires more extensive and definitive surgical treatment. However, DBE appears to play a role in the setting of rare complications related to tumours, such as small-bowel variceal bleeding, when radiological embolisation is not possible. There is limited literature on the role of small-bowel endoscopic techniques in the management of small-bowel NETs, and further data would be useful to determine their exact place in the diagnostic algorithm and therapeutic management of small-bowel NETs.

## Data Availability

Not applicable.
